# Infrared Spectroscopy as a Versatile Analytical Tool for the Quantitative Determination of Antioxidants in Agricultural Products, Foods and Plants

**DOI:** 10.3390/antiox4030482

**Published:** 2015-07-02

**Authors:** Daniel Cozzolino

**Affiliations:** School of Agriculture, Food and Wine, Faculty of Sciences, The University of Adelaide, Waite Campus, PMB 1 Glen Osmond SA, Adelaide, 5064, Australia; E-Mail: d.cozzolino@adelaide.edu.au; Tel.: +61-8-8313-6738

**Keywords:** antioxidants, near infrared, mid infrared, phenolic compounds, flavonols, plants, agricultural products

## Abstract

Spectroscopic methods provide with very useful qualitative and quantitative information about the biochemistry and chemistry of antioxidants. Near infrared (NIR) and mid infrared (MIR) spectroscopy are considered as powerful, fast, accurate and non-destructive analytical tools that can be considered as a replacement of traditional chemical analysis. In recent years, several reports can be found in the literature demonstrating the usefulness of these methods in the analysis of antioxidants in different organic matrices. This article reviews recent applications of infrared (NIR and MIR) spectroscopy in the analysis of antioxidant compounds in a wide range of samples such as agricultural products, foods and plants.

## 1. Introduction

The increasing market and consumer desire for quality of foods with positive health benefits has created a need for efficient and accurate analytical methods for the quantification of bioactive compounds and antioxidants in raw materials and finished products [1].

A large group of these bioactive compounds, antioxidants as well as secondary metabolites can be found in plants and agricultural products having a wide range of biological functions. For example, phenolic compounds are defined as secondary plant metabolites, which are important determinants in the sensory and nutritional quality of fruits, vegetables and other plants [2,3,4,5,6,7,8,9,10,11]. These compounds possess an aromatic ring bearing one or more hydroxyl groups and their structures may range from that of a simple phenolic molecule to that of a complex high-molecular mass polymer [2]. Phenolic compounds, one of the most widely occurring groups of phytochemicals, are of considerable physiological and morphological importance in plants. Natural occurring phenols have been reported to have excellent properties as food preservatives [2,3,4,5,6,7,8,9,10,11]. In the same way, flavonols and other plant metabolites (e.g., fatty acids) have also potent antioxidant activity [12,13].

Near infrared (NIR) and mid infrared (MIR) spectroscopy have been used to quantify several compounds having a diverse antioxidant capacity such as carotenoids, polyphenols, fatty acids and glucosinolates in a wide range of food commodities, for example, wine, dairy products, tea, fruit, vegetables, herbs, spices and cereals [1,14,15,16].

This article reviews recent applications of infrared (NIR and MIR) spectroscopy in the analysis of antioxidant compounds in agricultural products, foods and plants.

## 2. Current Analytical Methods

Several analytical methods have been or are used in the analysis of antioxidant compounds. Among them well established and highly efficient methods such as UV spectroscopy, high performance liquid chromatography (HPLC), gas chromatography (GC), liquid chromatography (LC), mass spectrometry (MS) and capillary electrophoresis (CE) have been routinely used for determination of bioactive and antioxidant compounds in several food products, herbs, plant extracts [14,15,16]. In recent years, GC coupled to MS and LC coupled to a diode array detector (DAD), electrochemical (ECD) and, in particular, tandem mass spectrometry (ESI-MS/MS) are the most widely used analytical techniques [17,18,19,20,21,22,23,24,25,26,27]. For example, GC methodologies provide higher resolution and sensitivity than LC methodologies but a laborious sample preparation stage is required, since they usually involve the isolation of metabolites by liquid-liquid extraction (LLE) or other extraction procedures followed by further derivatization [17,18,19,20,21,22,23,24,25,26,27]. In contrast, for LC analysis the sample preparation step is more simple and faster and provides a very sensitive method for the quantification of selected phenolic metabolites when coupled to MS/MS [17,18,19,20,21,22,23,24,25,26,27]. Although these methods provide with a high level of information about the chemistry of the compound measured, the sample requires different steps of pre-processing (e.g., extraction, purification, *etc.*) before and during the analysis.

## 3. Near and Mid Infrared Spectroscopy

Rapid, sensitive, and reliable analytical methods will be of benefit in order to determine the antioxidant capacity or value, to evaluate the contribution to the overall bioavailability of different antioxidant compounds and to allow the identification of biomarkers. In recent years, both near infrared (NIR) and mid infrared (MIR) spectroscopy with its intrinsic benefits such as being non-invasive, rapid, almost no necessary sample preparation, on-/inline measurements, have being able to determine physical and chemical parameters simultaneously, became a widely used analytical technique in the so called field of phyto-analytics and is included in pharmacopeia to an increasing extent [14,15,16,27,28].

Spectroscopic methods provide a technique that can be used in a wide range of biological samples without the need of extraction, which can often lead to degradation of the antioxidant components. Sample preparation time greatly decreases and analysis time is very short once a predictive model has been developed [14,15,16,27,28]. These methods can have a high degree of precision when applied to analysis of nutraceutical and antioxidant compounds concentration and antioxidant activity in foods [14,15,16,27,28]. Both NIR and MIR provide very useful qualitative and quantitative information of different classes of antioxidants due to its simplicity and low cost [14,15,16,27,28]. However, one of the main disadvantages of these methods is that they only give an estimation of the total phenolic content as they cannot separate individual compounds [14,15,16,27,28]. Table 1 summarises and compares the advantages and disadvantages of using spectroscopic and traditional analytical methods.

**Table 1:** Advantages and disadvantages of near infrared (NIR) and mid infrared (MIR) compared with traditional methods.

General Characteristics	NIR/MIR	Traditional Methods
**Sample pre-processing (extraction, dilution, grinding)**	Not required or minimal	Required
**Cost**	Relative low	Medium to high
**Speed of analysis**	high	Low to medium
**Need of standards**	Not required	Required
**Data analysis and interpretation**	Chemometrics is needed	Simple
**Quantitative analysis**	Yes (calibration with known samples)	Yes
**Qualitative analysis**	Yes	Yes

## 4. The Need of Multivariate Analytical Tools

The development and implementation of infrared (IR) methods for the analysis of antioxidants has been made possible by the development of multivariate data (MVA) methods and techniques in the so called field of chemometrics [14,15,16,27,28]. In the last 30 years, the use of chemometrics became more popular among scientist and analysts as computing power developed [14,15,16,27,28]. The incorporation of a computer as an integral part of the analysis also allowed the control of the instrument and the further identification of IR bands involved in the prediction [14,15,16,27,28]. Predictive methods (calibration) such as principal component (PCR) and partial least squares (PLS) regression are now widely used in the development of IR analytical methods for the prediction of antioxidant composition in a wide range of products [14,15,16,27,28]. In recent years, the analysis of antioxidants and antioxidant activity in food matrices using IR spectroscopy coupled with chemometric predictive models is now widely described by several authors as an alternative to wet chemistry, chromatographic determination and *in vitro* biochemical assays for assessment of antioxidant activity [14,15,16,27,28].

## 5. Examples of the Use of NIR and MIR Spectroscopy as Tools to Measure Antioxidants

### 5.1. Cereals, Small Grains and Sub-Products

The determination of total phenolic content and antioxidant capacity in coloured and white rice varieties from Thailand using Fourier transform infrared (FTMIR) spectroscopy was reported [29]. Dark glutinous rice had the highest level of antioxidant capacity and phytochemicals followed by red rice, brown rice, and white rice as indicated by the levels of phenolic content in rice samples [29]. Hierarchical cluster analysis based on selected principal components from a principal component analysis (PCA) discriminated rice varieties according to their color [29]. Partial least squares (PLS) regression models were developed and validated in order to predict total antioxidant capacity and total phenolic content of the rice varieties [29]. Results reported by the authors suggested that this technique is adequate for the quantification and prediction of antioxidant capacity and phenolic compound content of colored rice [29]. Phenolics and flavonoids are present in rice grains and they are associated with reduced risk of developing chronic diseases such as cardiovascular disease, type 2 diabetes, and some cancers [30]. The phenolic and flavonoid compounds in rice grain also contribute to its antioxidant activity [30]. A rapid and nondestructive predictive method based on NIR spectroscopy was proposed in order to measure the antioxidant capacity of rice [30]. The standard errors of prediction (SEP) reported by these authors were 47.1 and 45.9 mg gallic acid equivalent (GAE) for phenolic content [30]. Accurate performance for the prediction of antioxidant capacity (TEAC) with SEP of 0.28 mM was reported [30]. According to these authors the models based on NIR spectroscopy might be use for routine screening of a large number of rice samples in early generation screening in breeding programs [30].

Quinoa is a pseudo-cereal that is grown mainly in the Andes (South America) [31]. It is a functional food supplement and ingredient in the preparation of highly nutritious food [31]. The potential of NIR spectroscopy for the determination of vitamin E and antioxidant capacity in the quinoa as total phenol content (TPC), radical scavenging activity by DPPH (2,2-diphenyl-2-picryl-hydrazyl) and cupric reducing antioxidant capacity (CUPRAC) expressed as gallic acid equivalent (GAE) was described [31]. The multiple correlation coefficient (*R*^2^) and the standard prediction error corrected by bias (SEPc) reported by the authors were for the vitamin E (0.841 and 1.70 mg 100 g ^−1^), for the antioxidants TPC (0.947 and 0.08 mg GAE g ^−1^), DPPH radical (0.952 and 0.23 mg GAE g ^−1^) and for CUPRAC (0.623 and 0.21 mg GAE g ^−1^), respectively [31]. The results reported by the authors indicated that NIR spectroscopy provides an alternative for the determination of vitamin E and antioxidant properties of the quinoa, with a lower cost, higher speed and results comparable with the chemical reference methods currently in use [31].

The feasibility of measuring the antioxidant activity in dark soy sauce combining different chemometric methods and NIR spectroscopy was explored [32]. Different spectral intervals as well as nonlinear regression tools such as synergy interval (Si) PLS regression, kernel PLS (KPLS) and back propagation artificial neural network (BPANN) were reported by the authors [32]. The authors used cross-validation, and the performance of the final model was evaluated using R2 and the root mean square error of prediction (RMSEP) [32]. According to the authors the calibrations results based on Si-BPANN were superior to the other models, and the optimal result achieved a *R*^2^ > 0.97 and RMSEP = 0.0221 in the prediction set [32]. This work demonstrated that total antioxidant capacity in dark soy sauce could be measured by NIR spectroscopy technique, and Si-BPANN showed its superiority in model [32].

The application of NIR spectroscopy to the determination of the content of carotenoids in maize (*Zea mays*, L.), comparing the obtained data with those derived from HPLC determination of the extract obtained from the same samples was described [33,34]. The authors reported cross-validation results indicating good correlations between HPLC values and NIR spectroscopy [33]. The same authors have used similar approach to screen a large population of maize [34]. These results showed that NIR spectroscopy can be applied to determine the maize carotenoids in ground samples [33,34].

The estimation of tocopherol and phytosterol contents in sunflower seeds was also reported by combining NIR spectroscopy, HPLC, and gas chromatography [35]. For tocopherol content, the calibration data set ranged from 175 to 1005 mg·kg^−1^ oil (mean value around 5,106,140 mg·kg^−1^ oil), whereas for the phytosterol content, the calibration data set ranged from 180 to 470 mg 100 g^−1^ oil (mean value of 320,650 mg 100 g^−1^ oil) [35]. According to the authors relatively good correlation (*R*^2^ = 0.64) between predicted NIR data and reference values for the total tocopherol content, however poor correlation for the total phytosterol content (*R*^2^ = 0.27) was observed [35]. Overall, these results indicated that NIR spectroscopy could be useful to classify samples with high and low tocopherol content, although the estimation of phytosterol contents needs further investigation [35].

### 5.2. Fruits and Horticultural Products

The use of FTMIR spectroscopy was assessed to determine the bioactive compounds in kiwifruit as an indication of quality after extraction using methanol and ethyl acetate [36]. The contents of polyphenols, flavonoids, flavanols, and tannins, and the level of the antioxidant activity by 2,2-azino-bis (3-ethyl-benzothiazoline-6-sulfonic acid) diammonium salt, 1, 1-dipheny1-2-picrylhydrazyl, ferric-reducing/antioxidant power, and cupric reducing antioxidant capacity assays were determined and compared [36]. The authors found that the methanol extracts of kiwifruit showed significantly higher amounts of bioactive compounds and antioxidant activities than the ethyl acetate extracts [36]. The cultivar Bidan, in comparison with the classic Hayward, showed significantly higher bioactivity. The methods used are applicable for bioactivity determination, in general, for any food products [36].

The total phenolic contents and antioxidant activities of garlics from California, Oregon, Washington, and New York were determined using FTMIR spectroscopy [37]. The total phenolic content was quantified using Folin-Ciocalteu assay (FC) and the antioxidant activity was determined using DPPH assay, TROLOX equivalent antioxidant capacity (TEAC) assay, and ferric reducing antioxidant power (FRAP) [37]. Four independent PLS regression models were developed using spectra from 25 extracts and their corresponding FC, DPPH, TEAC, and FRAP reference values, yielding correlation coefficients > 0.95. The standard errors of calibration (SEC) and standard error of cross validation (SECV) were <1.45 (TEAC), 0.36 (FRAP), and 0.33 μmol TROLOX per g FW (DPPH) and 0.55 mg gallic acid per g FW (FC) [37].

Among different tropical fruits, durian (*Durio zibethinus* Murr.) is less known than mango (*Mangifera indica* L.) and avocado (*Persea americana*) [38]. It has been shown that durian, mango and avocado possessed high nutritional and bioactive properties, but these data were determined using different methods [38]. In order to obtain reliable results we investigated samples of durian, mango and avocado of the same stage of ripeness and unified methods were used for determination of the antioxidant potential [38]. The authors used lyophilised durian, mango and avocado samples sourced from Thailand and Israel [38]. The contents of crude protein, fat, carbohydrate, dietary fibre, total polyphenols, flavonoids, tannins and flavanols were determined by elemental analysis and UV spectroscopy [38]. The presence of polyphenols (flavonoids and phenolic acids) in the investigated samples was studied by FTIMIR spectroscopy and three-dimensional fluorometry [38]. Four complementary radical scavenging assays were used for antioxidant determination namely FRAP, 2,2-azino-bis (3-ethyl-benzothiazoline-6-sulfonic acid) diamonium salt (ABTS), DPPH and CUPRAC [38]. The analytical methods used (three-dimensional fluorescence, FTMIR spectroscopy, radical scavenging assays) were considered by the authors suitable for bioactivity determination of these fruits. The method proposed by the authors might be applicable for the bioactivity determination in phytochemical analysis in general [38].

The antioxidant content of fruits have been made them a desirable component of the human diet [39]. Several wet chemistry techniques, including the oxygen radical absorbance capacity (ORAC) assay, have been reported for measuring the antioxidant activities of fruit [39]. The use of FTMIR spectroscopy was investigated to measure fruit antioxidant activity. Flavonoid-rich extracts from blueberry, grape, and blackberry genotypes were obtained by methanol-water-formic acid (60:37:3, v/v/v) solvent and the total antioxidant capacities of the fruit extracts were determined by the ORAC assay [39]. Calibration models were developed using PLS regression with cross-validation. Good calibration models were reported by the authors where a *R*^2^ of 0.97 for antioxidant activity was obtained with satisfactory predictive ability RMSE of 5.35 [39]. The cross-validation models indicated good correlations, *R*^2^ of 0.94 between ORAC assay values and FTMIR estimates [39]. The ratio of the standard deviation of the data to the standard error of validation (RPD) values were above 5.0 for blueberry, grapes, and the combined extracts in the external validation set indicating that the calibration model was suitable for quantifying fruit extracts antioxidant activities [39]. This study showed that FTMIR would be suitable for rapidly measuring fruit extract antioxidant activity [39].

The use of low-cost screening techniques for nutritional compounds determination in food matrices is of increasing interest [40]. The potential of NIR spectroscopy for the prediction of antioxidant compounds in summer squash tissues collected since 2009–2012 was reported [40]. Modified MPLS regression was used to correlate spectral information and the different antioxidant compounds in the samples [40]. The *R*^2^ reported by the authors were for ascorbic acid (0.77 and 0.86), chlorophyll a (0.79 and 0.66), chlorophyll b (0.86 and 0.79) and total phenolic compounds (0.65 and 0.68) in exocarp and mesocarp tissues, respectively, supporting that NIR spectroscopy is able to predict in a rapid way these components for screening purposes [40]. The estimation of antioxidant components in tomato was reported by combining visible (VIS) and NIR spectroscopy [41]. The spectral ranges between 500 and 1000 nm were directly acquired on fruit puree of five different tomato varieties using a handheld instrument [41]. Calibration models were developed using PLS regression in order to predict lycopene and polyphenols [41].

Non-destructive measurement of vitamin C, total polyphenol and sugar content in more than 150 apple genotypes was reported using NIR spectroscopy [42]. According to the authors, results obtained using the least squares support vector machine (LS-SVM) regression method showed good to very good prediction performance [42]. Low standard error of prediction values, in addition to relatively high RPD values, demonstrated the precision of the models, especially for polyphenol and sugar content [42]. High RPD values (5.1 and 4.3 for polyphenol and sugar, respectively) indicated that an accurate classification of apples based on their content could be achieved [42]. NIR spectroscopy coupled with the multivariate calibration technique could be used to accurately measure the quality parameters of apples. The authors stated that in the case of breeding programs, NIR spectroscopy can be considered an interesting tool for sorting varieties according to a range of concentrations [42].

Calibrations for the discrimination of Chinese hawthorn (*Crataegus pinnatifida* Bge. var, major) fruits from three geographical regions as well as for the measurement of the total sugar, total acid, total phenolic content, and total antioxidant activity using NIR spectroscopy was also reported [43].

### 5.3. Olive Oil

Much analytical work has been published on the chemistry of extra virgin olive oil (EVOO) as a basis for the detection and quantitative analyses of the type and amount of adulteration with cheaper vegetable oils and deodorized olive oils [44]. The analysis and authentication of EVOO represent very challenging analytical chemical problems. A significant amount of literature on EVOO adulteration has depended on sophisticated statistical approaches that require analyses of large numbers of samples [44]. More effort is needed to exploit reliable chemical and instrumental methods that may not require so much statistical interpretation. Large assortments of methods have been used to determine lipid oxidation and oxidative stability and to evaluate the activity of the complex mixtures of phenolic antioxidants found in EVOO [44]. More reliable chemical methods are required in this field to obviate excessive dependence on rapid antiradical methods that provide no information on the protective properties of antioxidants [44]. The extensive literature on olive oil sensory tests, using many descriptors varying in different countries, should be supplemented by more precise GC analyses of volatile compounds influencing the aroma and flavours [44]. The combination of FTMIR spectroscopy with attenuated total reflectance was used to the determination of water content (WC), TPC and ABTS of virgin olive oils (VOO) and olive oils [45]. Calibration models were developed using PLS regression. Only oil samples with WC ranging from 289 to 1402 mg water/kg oil, with TPC from 46 to 877 mg gallic acid kg oil and with ABTS from 0 to 5.7 mmol TROLOX kg oil were considered for chemometric analysis [45]. According to the authors satisfactory LOD values using FTMIR spectroscopy were achieved 9.4 (mg/kg oil) for WC; 12.5 (mg gallic acid/kg oil) for TPC, and 0.76 (mmol TROLOX/kg oil) for ABTS [45]. Overall, FTMIR-ATR allowed the simultaneous evaluation of important quality parameters of VOO (WC, TPC and ABTS) [45]. According to the authors this approach represents an easy and convenient means for monitoring olive oil quality with the advantage of ease of operation, speed, no sample pre-treatment and no consumption of solvents [45]. The combination of FTMIR and ATR was also evaluated and reported for the determination of fatty acids (FFA) and peroxide value (PV) in virgin olive oil samples [46]. Calibration models were developed using PLS regression where the SEP reported by the authors were 3.0% for oleic acid, 0.5% for linoleic acid, 1.3% for SFA, 3.0% for MUFA, 0.3% for PUFA and 1.0 meq O(2) kg^−1^ for PV [46]. The proposed method provided results comparable to official procedures, with the advantages of being less expensive and fast [46].

### 5.4. Plant Extracts and Herbs

The concatenation of NIR and FTMIR-ATR spectroscopy were utilized to determine the antioxidant capacity of *Primulae floscum calycibus* samples [47]. The authors used FC, FRAP, DPPH, ABTS and CUPRAC assays as reference methods [47]. Cross and test-set validations were performed using PLS regression [47]. In general, NIR demonstrated advantages over ATR-MIR spectroscopy and resulted best for the ABTS assay *R*^2^ of 0.94, RPD of 4.66 in the validation set [47]. Also with ATR-MIR spectroscopy, the best prediction power was obtained for the ABTS assay *R*^2^ of 0.94, RPD of 4.10 in validation [47]. The feasibility of vibrational spectroscopy as a fast and simple tool to replace wet chemistry assays for the measurement of the antioxidant capacity of *Primulae floscum calycibus* samples was demonstrated [47].

The use of NIR spectroscopy has been developed for the simultaneous analyses of the chemical components and associated properties of mint tea (*Mentha haplocalyx* Brig.) [48]. Total polysaccharide content, total flavonoid content, total phenolic content, and total antioxidant activity were measured. The authors reported different calibration models and concluded that NIR spectroscopy can be considered an alternative method to measure antioxidant capacity in mint tea [48].

The ability of FTMIR spectroscopy was used to characterize the antioxidant activity of *Pereskia bleo* leaves by multivariate analysis [49]. Extracts were prepared in different solvents and the antioxidant activities were measured by 1,1-diphenyl-2-picrylhydrazyl assay [49]. A three component multivariate orthogonal PLS model with *R*^2^ of 0.88 and 0.86 was developed to correlate IR spectra with antioxidant activity and evaluated by internal cross-validation and a true external test [49]. The authors concluded that a successful orthogonal PLS model was developed using IR spectroscopy as a rapid method to predict antioxidant activity [49].

Two rapid screening methods, based in a colorimetric method employing aluminium chloride complexation and NIR spectroscopy, were evaluated for prediction of the mangiferin and xanthone contents of unfermented *Cyclopia subternata* plant materials [50]. The NIR spectroscopy calibration models developed for prediction of mangiferin (SEP = 0.21 g 100 g^−1^; *R*^2^ = 0.67) and xanthone (SEP = 0.27 g 100 g^−1^; *R*^2^ = 0.66) contents are suitable for screening purposes [50]. In order to improve the robustness of the NIR calibration models the data set were expanded to include data of unfermented *Cyclopia genistoides*, having higher xanthone content [50]. This procedure did not improve the NIR calibration models for the prediction of *C. subternata* samples, although the calibration was more robust for prediction of *C. genistoides* samples [50]. According to the authors using PCA and PLS discriminant analysis (PLS-DA) it was possible to clearly differentiate between the two species [50].

In recent years, some bioactive polysaccharides isolated from natural sources have attracted much attention in the field of biochemistry and pharmacology [51]. Of them, polysaccharides or their glycoconjugates were shown to exhibit multiple biological activities including anticarcinogenic, anticoagulant, immune stimulating and antioxidant capacity [51]. Pharmacotherapy using plant-derived substances can be currently regarded as a very promising future alternative to conventional therapy [51]. The advanced biotechnologies available today enable chemical investigation of well-defined bioactive plant components as sources of novel drugs [51]. The need for safer drugs without side effects has led to the use of natural ingredients with proven safety. Special interest is focused on plant polysaccharides from several plant species such as *Symphytum officinale* (comfrey), *Thymus pulegioides* (thyme), *Trigonella foenum-graecum* L. (fenugreek), *Tussilago farfara* L. (coltsfoot), *Hyssopus officinalis* (hyssop), *Althaea officinalis* L. (marshmallow) and *Equisetum arvense* L. (horsetail) [51]. The chemical structures of monosaccharides were analysed using FTMIR and Raman spectroscopies as well as cellulose acetate membrane electrophoresis (CAE). The dried plant samples were gently hydrolysed with sulphuric acid [51]. The presence of glucuronic acid, galacturonic acid, alginic acid, glucose, mannose and xylose in the hydrolysates of reference substances and non-defatted plant films was proved [51]. Individual bands in the IR region were selected to monitor the sugar content in medical plant cell walls and to confirm the identity of the analysed plants [51].

The effects of addition of rosemary and sage extracts on changes in iodine value (IV), PV and FFA content of refined, bleached and deodorized (RBD) palm olein during five days of deep fat frying of potato chips were monitored using FTMIR spectroscopy [52]. The monitoring was carried-out by comparing the results from wet chemical analysis and FTMIR spectroscopy. Results showed that rosemary and sage extracts could retard the deterioration of the oil during frying [52]. Both natural antioxidants were proven to lower the increases in PV and FFA content and the decrease in IV of RBD palm olein during frying [52]. The *R*^2^ of RBD palm olein during frying in relation to their chemical IV was 0.961, for PV 0.977 and for FFA content 0.978, respectively [52].

Radix *S. miltrorrhiza* belong to the same genus. *S. miltiorrhiza* var. alba and has a unique effectiveness for thromboangiitis besides therapeutical efficacy of *S. miltrorrhiza* [53]. This herb exhibits antioxidant activity, while its quality and efficacy also vary with geographic locations [53]. A method based in FT-NIR for the discrimination of geographical origin and evaluation of antioxidant activity of *S. miltiorrhiza* var. alba was reported [53]. The discrimination of geographical origin was achieved by using discriminant analysis and the accuracy was 100%. The use of PLS regression combined with different pre-treated methods were compared for the spectral pre-processing [53]. The *R*^2^, RMSEP and RPD values were 0.974, 0.163 mg·mL^−1^ and 2.66, respectively [53]. According to these authors the development of a NIR method might have a potential application to high-throughput screening of a great number of raw *S. miltiorrhiza* var. alba samples for antioxidant activity [53].

The comparison and hyphenation of ATR-MIR and NIR to quantify verbenalin and verbascoside in *Verbena officinalis* was also reported [54]. A new HPLC method as a reference was also established and validated by the authors [54]. Calibration statistics reported by the authors using ATR-MIR were for verbenalin *R*^2^ of 0.94, RPD of 4.23 and for verbascoside *R*^2^ of 0.93, RPD of 3.63 having advantages over NIR (verbenalin *R*^2^ of 0.91, RPD of 3.75; verbascoside *R*^2^ of 0.80, RPD of 2.35) [54]. Calibration models based on NIR spectroscopy were reported for the prediction of aspalathin, nothofagin and dihydrochalcone contents of dried, in green rooibos plant material [55]. These results showed that NIR spectroscopy can effectively predict the aspalathin content of dried, green rooibos with a SEP and *R*^2^ of 0.45 g 100 g^−1^ and 0.85, respectively and the dihydrochalcone content of rooibos with an SEP of 0.49 g 100 g^−1^ and *R*^2^ of 0.87 [55]. The authors also reported that after extending the aspalathin content range of the sample set, by adding varying amounts of a dried rooibos extract powder with high concentrations of aspalathin (15.95 g 100^−1^) and nothofagin (1.94 g 100 g^−1^) to some samples, slightly less accurate, but more robust, models were obtained for the aspalathin (SEP = 0.53 g 100 g^−1^; *R*^2^ = 0.87) and dihydrochalcone (SEP = 0.57 g 100 g^−1^); *R*^2^ = 0.88) contents [55]. The NIR calibration model developed for nothofagin content (SEP = 0.10 g 100 g^−1^; *R*^2^ = 0.71) (extended range: SEP = 0.10 g 100 g^−1^; *R*^2^ = 0.77) of dried, green rooibos is suitable for screening purposes [55].

### 5.5. Coffee and Tea

Early works in the use of NIR spectroscopy reported the use of this technique to predict the tea flavin content and moisture content of black tea [56] as well as to predict simultaneously the presence of alkaloids and phenolic substances in green tea leaves [56]. More recently, the use of NIR spectroscopy was evaluated for the quantitative analysis of total antioxidant capacity of green tea [57]. The simultaneous analysis of main catechins in tea such as epicatechin (EC), epicatechin-3-gallate, epigallocatechin, epigallocatechin-3-gallate (EGCG) using FT-NIR spectroscopy was also reported [58]. The combination of NIR spectroscopy with PLS regression was reported to predict the content of caffeine, EGCG, EC and total antioxidant capacity in green tea [59]. For the determination of the total antioxidant capacity, TROLOX equivalent antioxidant capacity (TEAC) method was used by the authors [59]. Models based on PLS regression that gave the lowest RMSECV for the training set were selected by the authors [59]. The correlation coefficient (R) between the predicted and the reference results for the test set is used as an evaluation parameter for the models for the TEAC results R of 0.90 for the model with the whole leaves, R of 0.86 for the model with the powdered leaves were reported [59]. The caffeine prediction model has an R of 0.96 for the whole leaves and R of 0.93 for the ground leaves [59]. The R for the EGCG and the EC content models was 0.83 and 0.44, respectively [59].

Epigallocatechin-3-gallate (EGCG) is credited with the majority of the health benefits associated with green tea consumption and has a high economic and medicinal value [60]. The feasibility of using different variable selection approaches in FT-NIR spectroscopy for the quantitative determination of EGCG in green tea was investigated [60]. The calibration methods reported by the authors included interval partial least squares (iPLS), SiPLS, and genetic algorithm (GA) optimization combined with SiPLS (SiPLS-GA) were applied to select the most efficient spectral variables that provided the lowest prediction error [60]. The performance of the final model was evaluated according to the RMSEP and *R*^2^ for the prediction set [60]. The results showed that the SiPLS-GA model obtained the best results with *R*^2^ of 0.97 and RMSEP of 032 [60]. A PCR model was also reported for the prediction of total antioxidant capacity in green tea using NIR spectroscopy. The RMSEP values of the final model were comparable to the precision of the reference method [61].

Spent coffee grounds are a significant residue produced annually worldwide [62]. This by-product contains high levels of bioactive compounds, such as chlorogenic acid and flavonoids that have recognized antioxidant properties [62]. The use of FT-NIR spectroscopy was reported to determine the antioxidant capacity, TPC and total flavonoid contents of several spent coffee samples. The antioxidant capacity of the samples was determined by means of the ABTS assay, using the direct procedure that measures both soluble and insoluble antioxidant fractions and by the indirect approach that assesses the antioxidants present in ethanolic extracts [62]. The authors reported an *R*^2^ of 0.93, 0.96, 0.95 and 0.95 for antioxidant capacity, antioxidant capacity of ethanolic extracts, total flavonoid and TPC, respectively [62]. The range error ratio (RER) was higher than 13 for all models (higher than 16 for three of them) [62]. Overall, the results confirmed that NIR spectroscopy is a promising technique for routine assessment of these parameters in spent coffee grounds samples and is a viable and advantageous alternative to chemical procedures involving laborious extractions [62]. The measurement of antioxidant activity in green tea using NIR spectroscopy was reported [63]. Different linear and nonlinear regressions tools such as PLS, BPANN, and SVM were also tested in order to optimise the calibration model [63]. The optimum results were achieved using SVM regression were a RMSEP of 0.021 and R of 0.96 in the prediction set was reported by the authors [63]. The overall results demonstrated that NIR spectroscopy coupled with the SVM regression has the potential to measure antioxidant capacity in green tea samples [63].

### 5.6. Wine

The total antioxidant activity in red wine samples was reported using FTMIR spectroscopy [64,65]. The PLS models based in cross validation showed a good predictive capacity (*r* = 0.85) of the antioxidant activity of red wines [64,65]. The authors concluded that FTIR spectroscopy is a promising technique to rapidly provide information on total antioxidant activity of red wines and has a high potential to be implemented for rapid screening of several wine samples [64,65].

### 5.7. Honey

The measurement of antioxidant capacity in honey was reported using NIR spectroscopic combined with MPLS as regression method [66]. The authors reported calibration models for the estimation of TPC, flavonoids, vitamin C, DPPH, oxidation index and copper [66]. Overall, the authors concluded that NIR spectroscopy can be considered as a rapid tool for the non-destructive measurement of antioxidant constitutes (TPC, flavonoids, vitamin C, copper and DPPH) in honey [66].

## 6. Conclusions

As fast and easy-to-operate technique, IR spectroscopy has already gained wide acceptance for the routine analysis of antioxidants in a wide range of foods, plants and agricultural products. Although spectroscopy generally cannot measure molecules with low concentration, the indirect effects of such differences in the whole matrix of the sample can be observed or assessed in the IR spectrum of a given sample (fingerprint). This fingerprint, with the application of chemometric techniques (e.g., principal component analysis or discriminant analysis), can be used to elucidate particular compositional characteristics not easily detected by traditional targeted chemical analysis is one of the main advantages.

Calibration development is, by far, the critical aspect in order to develop a successful method based in IR spectroscopy. This step requires a high level of expertise, particularly in chemometrics or multivariate data analysis in order to make an IR application successful.

Spectroscopic methods based in IR also offer potential savings for the industry and research in aspects related with the reduction of analysis time and cost, as well as with the environmentally friendly nature of the technology, positioned IR spectroscopy as a very attractive technique. It is clear that the breadth of applications based on IR spectroscopy for the measurement of antioxidants and antioxidant capacity in a wide range of samples, either in routine use or under developed is showing no sign of diminishing.

One of the most important critical aspects of the development of IR spectroscopy is the need for appropriate training, for example routine analyses can be performed by analysts with a high school education, although knowledge of the chemistry of the sample material will be useful.

However, the lack of academic education and formation in relevant aspects of molecular spectroscopy and chemometrics are still a barrier for the wide spread acceptance of this technology.
